# Elastoplastic Analysis of Plane Stress Problems for Porous Plastic Material

**DOI:** 10.3390/ma19040761

**Published:** 2026-02-15

**Authors:** Jiaxing Zeng, Jianxiong Liu, Tiansu Li, Xiangming Wan, Youdong Jia

**Affiliations:** 1 Faculty of Mechanical and Electrical Engineering, Kunming University of Science and Technology, Kunming 650500, China; zengjiaxing@stu.kust.edu.cn (J.Z.); wanxiangming199@163.com (X.W.); jiayoudong@stu.kust.edu.cn (Y.J.); 2 College of Surveying, Mapping and Information Engineering, West Yunnan University of Applied Sciences, Dali 671000, China; tiansu_li@126.com

**Keywords:** two-parameter yield criterion, porous plastic material, mesoscale damage model, plane stress problems, elastoplastic analysis

## Abstract

Considering the impact of void damage on the mechanical properties of materials, based on the two-parameter yield criterion, combined with the associated flow rule and the upper bound theorem, the void volume fraction is introduced into the macroscopic yield function, resulting in a mesoscale damage model. Two material parameters in the model are defined using yield strength and Poisson’s ratio, respectively. The yield surface of the model is presented for different void volume fractions and Poisson’s ratios. Using the mesoscale damage model, combined with the positive flow rule, the constitutive relationship of the material is established, and an elastoplastic analysis is performed for axisymmetric plane stress problems. Under the Prager hypothesis, a set of differential equations is derived to solve the problem, yielding numerical solutions. The influence of void volume fraction on the stress field and displacement field is qualitatively discussed. The research results show that when the void volume fraction is constant, the closer to the void opening, the larger the absolute value of radial stress and displacement, and the faster the material flows, with the material reaching the yield state first. As the void volume fraction increases, both the absolute value of radial stress and displacement decrease relatively. In contrast, the change in circumferential stress is relatively small, but the patterns of the numerical result curves tend to be consistent.

## 1. Introduction

Metal materials are widely used in aerospace, rail transit, megaships, pressure vessels, and other critical fields due to their excellent mechanical properties. Therefore, the deformation and failure behavior of metal materials in service has attracted much attention. The thin plate structure is one of the most common structures in the application of metal materials in various fields, such as wings in aerospace and decks in marine engineering. The deformation behavior of such a thin plate structure under in-plane load can be regarded as a plane stress problem, so the analysis of the plane stress problem has always been a hot topic in scientific research and engineering practice. Through the analysis of a plane stress problem, the stress distribution and deformation of this kind of structure under load can be accurately predicted, which provides an important theoretical basis for the engineering application of metal materials.

Using the plane stress hypothesis, the study of macrofracture mechanics plays a fundamental role in analyzing crack propagation. Kirsch [1] obtained the analytical solution of an infinite plate with a circular hole under tension by using the general polar coordinate solution of the plane problem. Inglis [2] solved the plane problem of the tensile stress of an infinite plate with an elliptical hole in the elliptic coordinate system. Westergaard [3] used the stress function method to solve the tensile problem of an infinite plate with an ideal crack. Irwin et al. [4] analyzed the crack tip stress field of an elliptical crack in an infinite plate under plane stress and pointed out the effect of stress intensity factors on crack growth. Guo et al. [5] used the theory of compressible plasticity to analyze the propagation of plane stress cracks in metal foams.

The research shows that the properties of the material and the yield criterion used have an impact on the analysis of the plane stress problem. Based on the yield criterion (MVM criterion) proposed by Tschoegl, Tang et al. [6] established the constitutive relationship of the material by using the associated flow rule, and conducted an elastoplastic analysis of the plane stress axisymmetric problems commonly seen in engineering. Bijak-zochowski et al. [7] considered the effects of strain hardening, yield criterion, and stress/strain state of materials, and studied the plastic zone, deformation, stress, and residual stress of plane indenters with different shapes in elastoplastic contact with semi-infinite bodies. Zhou et al. [8] used the orthotropic quadratic yield function to analyze the deformation of plastic anisotropic and strain hardening materials under axisymmetric plane stress, and gave a general analytical solution to such problems. Li et al. [9] considered the anisotropic strengthening of metal materials in service, and gave the stress field and stress characteristic field of the plane stress problem of orthotropic materials based on hill yield criterion, and obtained the parameters determining the characteristics of the characteristic curve by using the stress differential equilibrium equation. Liu et al. [10] considered the essence of metal plastic deformation and derived the criteria for determining the stability of metal plastic deformation of isotropic and anisotropic materials under plane stress based on the plastic deformation theory. Li et al. [11] used strict physical concepts and the trial and error method to derive strict analytical solutions of two-dimensional plane problems of ideal plastic materials under axisymmetric conditions. Liu et al. [12] used the basic equation of the plane stress problem, combined with the single-parameter plastic model, the orthogonal flow rule, and the linear strain hardening law, and obtained the analytical solution of the stress and displacement of the thermoplastic composite plane curved beam under the action of bending moment. Roman [13] considered the plane stress situation; adopted the Cowin–Nunziato linear model, which can describe the static equilibrium of porous elastomer; gave the general solution of the plane stress problem according to the harmonic function and Helmholtz equation; proposed an algorithm to solve the corresponding boundary value problem by using the basic solution; and obtained the approximate solution of the boundary value problem in the square region with a circular hole in the center. Chen et al. [14] proposed a mixed formula for the plane stress problem, derived the equilibrium equation of the out-of-plane normal strain by using the variational principle, and calculated the non-uniform stress field problem by using the elastic and plastic models, which showed the effectiveness and accuracy of the method. Lyamina [15] used the plastic flow theory and, based on the isotropic yield criterion, derived the general solution of axisymmetric elastoplastic problems under plane stress conditions.

For the plane stress problem in engineering, experimental research can effectively prove the accuracy of the theoretical analysis. Li et al. [16] used the displacement solution to derive the general solution of the displacement solution of the axisymmetric plane stress problem in the plastic state, and based on this, analyzed the variation law of the blank height and outer diameter during the hydraulic bulging process of the generator retaining ring. Qin et al. [17] discussed the stress distribution in the flange area of axisymmetric deep drawing under the assumption of plane stress, and deduced the wrinkling instability condition and the calculation model of critical blank holder force of axisymmetric deep drawing based on the principle of energy method. Giginyak and Bulakh [18] used experimental methods to study the deformation process and damage development of pre-deformed 10GN2MFA heat-resistant steel under plane stress cyclic loading. Qin et al. [19] aimed at the general axisymmetric forming problem, based on the assumption of plane stress and thin plate theory, using the incremental theoretical solution, and obtained the strain distribution in the flange area and the die fillet area of cylindrical and conical parts in the deep drawing process. Woelke [20] considered the influence of a micro-fracture mechanism on material failure, based on the shear modified Gurson model and its implementation in the generalized plane stress state, proposed a plastic model, and verified the feasibility of the model by comparing the finite element simulation using a shell element and the three-point bending test of a high-strength steel beam. Givoli [21] made an asymptotic analysis of the linear elastic thin plate made of uniform anisotropic materials under the assumption of plane stress, and obtained the second-order solution, which was proved to be the exact solution of the 3D problem of isotropic materials in special cases. Mu et al. [22] introduced the principal stress axis direction into the anisotropy parameter and established a modified model of the Hill 48 yield function suitable for a plane stress state. The model can accurately capture the directional yield stress and yield trajectory, and can effectively predict the anisotropic behavior of aluminum alloys and other materials. Bulakh [23] and others considered the influence of stress state types on the damage development of materials, used the LM hardness method to evaluate the damage level in materials, and studied the damage development of 10GN2MFA and 15Kh2MFA heat-resistant steel under plane stress conditions. The results showed that this method could quickly evaluate the damage degree of heat-resistant steel tubular structures in the process of use. Zhu et al. [24] proposed a uniform anisotropic hardening (HAH) model under plane stress for the plane stress problem of sheet metal forming, and verified the feasibility of the model by analyzing the stress–strain response of DP780 and EDDQ steel plates under uniaxial tensile test.

Although many studies have been conducted on plane stress problems, most of them were carried out from the macro perspective, thus ignoring the influence of micro-damage on the mechanical properties of materials in the process of deformation. Considering the influence of micro-damage evolution on the mechanical properties of materials, this paper adopts the two-parameter yield criterion, introduces the micro-damage parameter (void volume fraction f) into the macro yield function through theoretical analysis, and analyzes the influence of different parameters on the yield surface. Based on the new yield function, the constitutive equation of the general plane stress problem is established. The elastic–plastic analysis of an infinite plate with a hole under the pressure of a parallel plate at the hole is carried out. The differential equations of stress, displacement, and plastic flow factors in the process of elastic–plastic deformation are derived, and the numerical solution is found. The influence of different void volume fractions on the field is analyzed.

## 2. Establishment of Meso-Damage Model

### 2.1. The Plastic Dissipation

The existing research results show that the process of plastic deformation to fracture failure of metal materials is affected by the nucleation, growth, and polymerization of internal micropores, and the evolution process of micropores is affected by hydrostatic pressure. Therefore, it is necessary to derive the yield function that can describe the microscopic mechanism based on the existing macroscopic yield criterion when analyzing the mechanical behavior of material deformation. Firstly, it is assumed that the yield condition of the matrix material obeys the two-parameter parabolic function proposed by Christensen [25,26]

(1) $$F\;=\frac{3}{2}(1+\alpha )s_{ij}s_{ij}+\alpha K\sigma_{kk}-K^{2}=0$$

where *K* and *α* are material parameters.

According to the associated flow rule, the strain rate is

(2) $${\dot{\varepsilon }}_{ij}=\lambda \frac{\partial F}{\partial \sigma_{ij}}=\lambda [3\left(1+\alpha \right)s_{ij}+\alpha K\delta_{ij}]$$

Therefore, the strain rate deviatoric tensor ${\dot{e}}_{ij}$ and the strain rate spherical tensor ${\dot{\varepsilon }}_{kk}$ can be expressed as

(3) $${\dot{e}}_{ij}=3\lambda \left(1+\alpha \right)s_{ij}$$

(4) $${\dot{\varepsilon }}_{kk}=3\lambda \alpha K$$

Therefore, λ can be expressed as

(5) $$\lambda =\frac{{\dot{e}}_{ij}}{3\left(1+\alpha \right)s_{ij}}=\frac{1}{2\left(1+\alpha \right)}\frac{\dot{\bar{\varepsilon }}}{\bar{\sigma }}$$

where $\bar{\sigma }$ and $\dot{\bar{\varepsilon }}$ are Mises equivalent stress and equivalent strain rate, respectively.

Substitute Equation (5) into Equation (4) to obtain

(6) $${\dot{\varepsilon }}_{kk}=\frac{3\alpha K}{2\left(1+\alpha \right)}\frac{\dot{\bar{\varepsilon }}}{\bar{\sigma }}$$

So

(7) $$\bar{\sigma }=\frac{3\alpha K}{2\left(1+\alpha \right)}\frac{\dot{\bar{\varepsilon }}}{{\dot{\varepsilon }}_{kk}}$$

Substitute Equation (7) into Equation (1) to obtain

(8) $$\sigma_{kk}=\frac{K}{\alpha }-\frac{9\alpha K}{4\left(1+\alpha \right)}\frac{{\dot{\bar{\varepsilon }}}^{2}}{{{\dot{\varepsilon }}_{kk}}^{2}}$$

According to the plastic dissipation power calculation formula

(9) $$\dot{w}=\sigma_{ij}{\dot{\varepsilon }}_{ij}=\left(s_{ij}+\frac{1}{3}\sigma_{kk}\delta_{ij}\right)\lambda \left[3\left(1+\alpha \right)s_{ij}+\alpha K\delta_{ij}\right]$$

and substituting Equations (5)–(8) into Equation (9), the plastic dissipation power expression can be obtained as

(10) $$\dot{w}=A\frac{{\dot{\bar{\varepsilon }}}^{2}}{{\dot{\varepsilon }}_{kk}}+B{\dot{\varepsilon }}_{kk}$$

where $A=\frac{3\alpha K}{4\left(1+\alpha \right)}$, $B=\frac{K}{3\alpha }$.

### 2.2. Meso-Strain Rate

To connect the microscopic velocity field with the macroscopic field, it is necessary to derive the simple velocity field of the analytical yield function. For a spherical cavity, the meso-strain rate field is decomposed into the volume change part ${\dot{\varepsilon }}_{ij}^{V}$ and the shape change part ${\dot{\varepsilon }}_{ij}^{S}$, i.e.,

(11) $${\dot{\varepsilon }}_{ij}={\dot{\varepsilon }}_{ij}^{V}+{\dot{\varepsilon }}_{ij}^{S}$$

Similarly, the velocity field is decomposed into two parts

(12) $$v_{i}=v_{i}^{V}+v_{i}^{S}$$

that is, the volume change part $v_{i}^{V}$ under the condition of unchanged shape and the shape change part $v_{i}^{S}$ under the condition of unchanged volume.

Assuming that the spherical hole is still spherical after deformation during the whole deformation process, the shape will not change; only the volume will change. At this time, $v_{\theta }^{V}=v_{\phi }^{V}=0$.

The meso-volume strain rate is expressed as

(13) $${\dot{\varepsilon }}_{kk}={\dot{\varepsilon }}_{rr}+{\dot{\varepsilon }}_{\theta \theta }+{\dot{\varepsilon }}_{\phi \phi }=a_{0}$$

The relationship between strain rate and speed is

(14) $${\dot{\varepsilon }}_{rr}=\frac{\partial v_{r}^{V}}{\partial r},{\dot{\varepsilon }}_{\theta \theta }=\frac{v_{r}^{V}}{r},{\dot{\varepsilon }}_{\phi \phi }=\frac{v_{r}^{V}}{r}$$

Therefore, Equation (13) can be written as

(15) $${\dot{\varepsilon }}_{kk}=a_{0}=\frac{\partial v_{r}^{V}}{\partial r}+2\frac{v_{r}^{V}}{r}$$

This deformation mode is the same as the displacement mode of elastic deformation of a spherical cavity. The relationship between the micro-velocity field at the cell surface s and the macro velocity field in Cartesian coordinate system is as follows:

(16) $$v_{i}{|}_{S}={\dot{E}}_{ij}X_{j}{|}_{S}$$

where ${\dot{E}}_{ij}$ is the macro strain rate. From $v_{\theta }=v_{\phi }=0$, then ${\dot{E}}_{11}={\dot{E}}_{33}$, so at the boundary *r* = *b*, there is

(17) $$v_{r}=b{\dot{E}}_{11}=\frac{b}{3}{\dot{E}}_{kk}$$

Substitute Equation (17) into Equation (15) to obtain

(18) $$v_{r}^{V}=\frac{1}{3}\left({\dot{E}}_{kk}-a_{0}\right)\frac{b^{3}}{r^{2}}+\frac{a_{0}}{3}r$$

Then, according to Equation (14), it can be obtained that

(19) $${\dot{\varepsilon }}_{rr}=\frac{\partial v_{r}^{V}}{\partial r}=-\frac{2}{3}\left({\dot{E}}_{kk}-a_{0}\right)\frac{b^{3}}{r^{3}}+\frac{a_{0}}{3}$$

(20) $${\dot{\varepsilon }}_{\theta \theta }={\dot{\varepsilon }}_{\phi \phi }=\frac{v_{r}^{V}}{r}=\frac{1}{3}\left({\dot{E}}_{kk}-a_{0}\right)\frac{b^{3}}{r^{3}}+\frac{a_{0}}{3}$$

Referring to Rice and Tracey’s hypothesis [27], the velocity field related to the shape change has little effect on the growth rate of the hole; that is, the spherical hole is still spherical after deformation. So, ${\dot{\varepsilon }}_{ij}^{S}$ caused by the shape change is equal to the macro-strain rate deviatoric tensor ${\dot{E}}_{ij}^{\prime }$, and the micro-strain rate is

(21) $${\dot{\varepsilon }}_{ij}={\dot{E}}_{ij}^{\prime }+\frac{1}{3}\left({\dot{E}}_{kk}-a_{0}\right)h_{ij}+\frac{1}{3}a_{0}\delta_{ij}$$

In Cartesian coordinates

(22) $$h_{ij}=\left(\delta_{ij}-3n_{i}n_{j}\right){\left(\frac{b}{r}\right)}^{3},n_{i}=\frac{x_{i}}{r}$$

(23) $$h_{rr}=-2{\left(\frac{b}{r}\right)}^{3}={-2h}_{\theta \theta }={-2h}_{\phi \phi }$$

(24) $$h_{ij}{|}_{i\neq j}=0,h_{ij}h_{ij}=6{\left(\frac{b}{r}\right)}^{6}$$

Therefore, it can be concluded that

(25) $${\dot{e}}_{ij}={\dot{\varepsilon }}_{ij}-\frac{1}{3}{\dot{\varepsilon }}_{kk}\delta_{ij}={\dot{E}}_{ij}^{\prime }+\frac{1}{3}\left({\dot{E}}_{kk}-a_{0}\right)h_{ij}$$

(26) $${\dot{\bar{\varepsilon }}}^{2}=\frac{2}{3}{\dot{e}}_{ij}{\dot{e}}_{ij}={\dot{\bar{E}}}^{2}+\frac{4}{9}{\dot{E}}_{ij}^{\prime }\left({\dot{E}}_{kk}-a_{0}\right)h_{ij}+\frac{4}{9}{\left({\dot{E}}_{kk}-a_{0}\right)}^{2}{\left(\frac{b}{r}\right)}^{6}$$

According to the principle of macro-micro-power equivalence

(27) $$\int_{V}\sigma_{ij}{\dot{\varepsilon }}_{ij}dV=\Sigma_{ij}{\dot{E}}_{ij}V$$

where $V=4\pi b^{3}/3$ is the volume of the unit cell, from which the deformation power of the macro unit cell can be obtained as

(28) $$\dot{W}=\Sigma_{ij}{\dot{E}}_{ij}=\frac{1}{V}\int_{V}\sigma_{ij}{\dot{\varepsilon }}_{ij}dV$$

According to the upper bound theorem, the real velocity fields $v_{i}$ and ${\dot{\varepsilon }}_{ij}$ make $\dot{W}$ obtain the minimum value

(29) $$\frac{\partial \dot{W}}{\partial a_{0}}=0$$

Meanwhile

(30) $$\frac{1}{V}\int_{V}dV=\frac{3}{4\pi b^{3}}\int_{r=a}^{r=b}\int_{\theta =0}^{\theta =\pi }\int_{\phi =0}^{\phi =2\pi }r^{2}\sin\theta drd\theta d\phi$$

(31) $$\frac{1}{V}\int_{V}h_{ij}M_{ij}dV=0$$

where $M_{ij}$ is a function of *r*, which can be obtained by combining Equations (10), (26), (28), and (29)

(32) $$\frac{3}{b^{3}}\int_{b}^{a}\left\{B-\frac{A}{{a_{0}}^{2}}\left[{\dot{\bar{E}}}^{2}+\frac{4}{9}\left({{\dot{E}}_{kk}}^{2}-{a_{0}}^{2}\right){\left(\frac{b}{r}\right)}^{6}\right]\right\}r^{2}dr=0$$

available after sorting

(33) $$a_{0}={\left(\frac{9fA{\dot{\bar{E}}}^{2}+4A{{\dot{E}}_{kk}}^{2}}{9fB+4A}\right)}^{1/2}$$

It can be found that when *f* = 0, $a_{0}={\dot{E}}_{kk}$.

### 2.3. Macro-Stress and Yield Function

After the macro- and micro-analysis of the same cell with a spherical hole in the center, the plastic power of the two layers is equal. The macro-stress state can be obtained by calculating the partial derivative of the plastic power with respect to the macro-strain rate, and then the yield condition of the macro-layer material can be obtained. According to the power equivalence between macro and micro, the macro-stress can be expressed by the micro-stress and -strain rate

(34) $$\Sigma_{ij}=\frac{\partial \dot{W}}{\partial {\dot{E}}_{ij}}=\frac{1}{V}\int_{V}\sigma_{kl}\frac{\partial {\dot{\varepsilon }}_{kl}}{\partial {\dot{E}}_{ij}}dV$$

Combined with the expression of the meso-strain rate (21), the expression of macro-stress is

(35) $$\Sigma_{ij}^{\prime }=\frac{1}{V}\int_{V}\sigma_{ij}^{\prime }dV=\frac{1}{V}\int_{V}s_{ij}dV$$

(36) $$\Sigma_{nn}=\frac{1}{V}\int_{V}\sigma_{kl}^{\prime }h_{kl}dV=\frac{1}{V}\int_{V}\sigma_{ij}^{\prime }h_{ij}dV$$

According to Formulas (5), (7), and (25), the

(37) $$s_{ij}=\frac{\alpha K}{(1+\alpha ){\dot{\varepsilon }}_{kk}}[{\dot{E}}_{ij}^{\prime }+\frac{1}{3}\left({\dot{E}}_{kk}-a_{0}\right)h_{ij}]$$

Substituting Equation (37) into Equations (35) and (36), respectively, we can find

(38) $$\Sigma_{ij}^{\prime }=\frac{\alpha K}{\left(1+\alpha \right)a_{0}}{\dot{E}}_{ij}^{\prime }(1-f)$$

(39) $$\Sigma_{nn}=\frac{8A\left({\dot{E}}_{kk}-a_{0}\right)(1-f)}{3a_{0}f}$$

where the hole volume fractions $f=a^{3}/b^{3}$. Therefore, the macroscopic equivalent stress is

(40) $$\Sigma_{eq}=\sqrt{\frac{3}{2}\Sigma_{ij}^{\prime }\Sigma_{ij}^{\prime }}=\frac{2A\dot{\bar{E}}(1-f)}{a_{0}}$$

By substituting the expression of $a_{0}$ into Equations (39) and (40), the macroscopic yield function containing void volume fraction can be obtained as

(41) $${\Sigma_{eq}}^{2}+\frac{f}{4}{\Sigma_{nn}}^{2}+\frac{\alpha K(1-f)}{1+\alpha }\Sigma_{nn}-\frac{K^{2}}{1+\alpha }{\left(1-f\right)}^{2}=0$$

**Figure 1:**
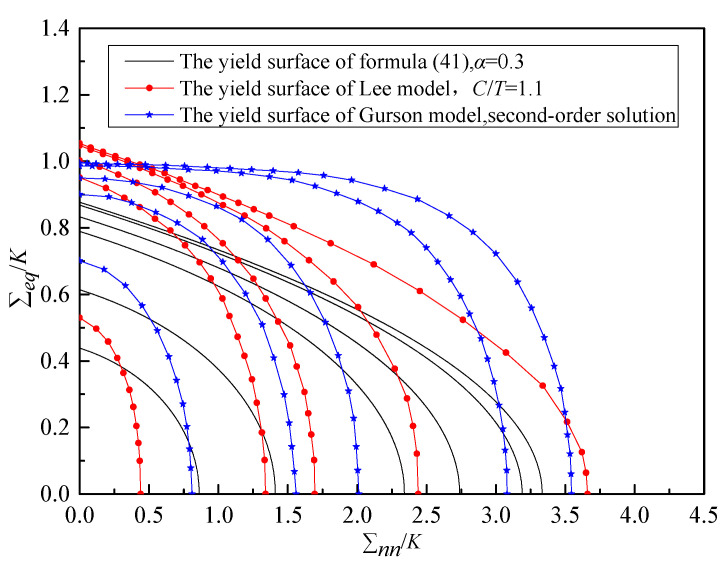
Yield surface comparison for different model under different *f*. The *f* values are (from right to left) as follows: Formula (41), *f* = 0, 0.01, 0.05, 0.1, 0.3, 0.5; Lee model, *f* = 0, 0.01, 0.05, 0.1, 0.5; Gurson model, *f* = 0.005, 0.01, 0.05, 0.1, 0.3.

**Figure 2:**
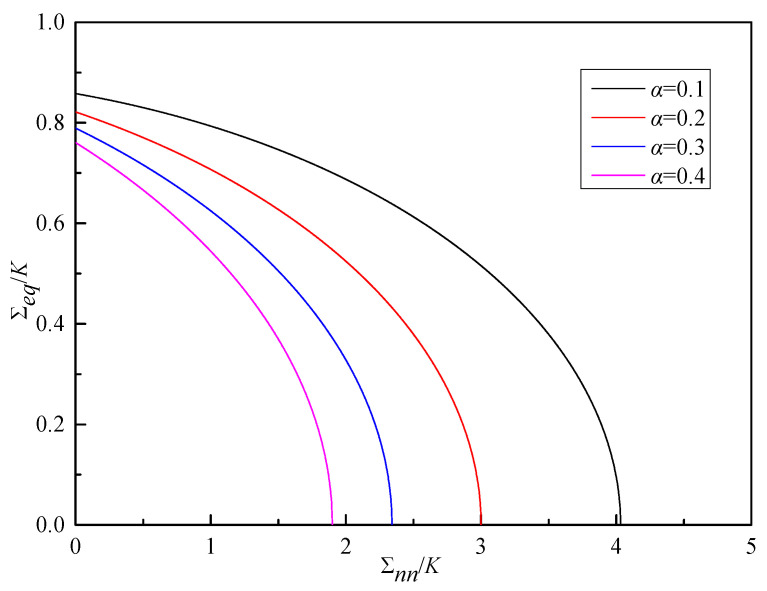
Yield surface for Formula (41) under different *α* when *f* = 0.1.

If the void damage (*f* = 0) is not considered, the yield function Equation (41) can be reduced to the initial form of Equation (1). If the absolute values of the uniaxial tensile yield strength $\sigma_{s}^{T}$ and the uniaxial compressive yield strength $\sigma_{s}^{C}$ of the material are introduced to define the forms of material parameters *α* and *K*, respectively, the above yield function can be transformed into the Lee model [28]. Mises’ yield criterion takes the uniaxial tensile yield strength of the material as the judgment standard for material yield, and relies on the influence of the deviatoric stress tensor on material deformation, while Poisson’s ratio is directly related to the volume deformation of the material. Therefore, this paper attempts to use the material yield strength and Poisson’s ratio to separately define the material parameters *K* and *α*, and gives the yield surfaces of Formula (41) with different *f* and α values, as shown in Figure 1 and Figure 2.

As can be seen from Figure 1, under a given material parameter *α*, as the void volume fraction *f* increases, the area of the yield surface envelope decreases, indicating that the material is more prone to yield. Furthermore, as f increases, the influence of the term ${\Sigma_{nn}}^{2}$ gradually intensifies, significantly deviating from the case of *f* = 0. This demonstrates the significant impact of the void volume fraction on the stress distribution in the material, which is similar to the conclusions drawn from the Lee model and Guron model in Figure 1 [28,29]. However, due to differences in the underlying models and parameters, there are certain differences in their yield surfaces at the same void volume fraction, leading to variations in the area of the yield surface envelope among the three models. As can be seen from Figure 2, for a given *f* value, *α* also has a significant impact on the yield of the material. As the value of *α* increases, the yield surface of the material gradually contracts.

## 3. Elastoplastic Solution for Plane Stress Axisymmetric Problems

### 3.1. Yield Condition and Constitutive Equation

Elastoplastic analysis is carried out for general plane stress problems. Firstly, the constitutive equation is given according to the yield condition. For the convenience of expression, the following stresses and strains are represented in lowercase letters as macroscopic physical quantities, and the macroscopic yield condition of the material can be given by Equation (41), which can be written as

(42) $$F\left(\sigma_{ij}\right)=\frac{3}{2}s_{ij}s_{ij}+\frac{f}{4}{\sigma_{kk}}^{2}+\frac{\alpha K(1-f)}{1+\alpha }\sigma_{kk}-\frac{K^{2}}{1+\alpha }{\left(1-f\right)}^{2}=0$$

In the axisymmetric problem of plane stress, $\sigma_{r}$ and $\sigma_{\theta }$ are not zero, but $\sigma_{z}=0$, and the shear stress component is also zero; then,

(43) $$\frac{3}{2}s_{ij}s_{ij}=\frac{1}{2}\left[{\left(\sigma_{r}-\sigma_{\theta }\right)}^{2}+{\sigma_{r}}^{2}+{\sigma_{\theta }}^{2}\right]={\sigma_{r}}^{2}+{\sigma_{\theta }}^{2}-\sigma_{r}\sigma_{\theta }$$

(44) $$\sigma_{kk}=\sigma_{r}+\sigma_{\theta }$$

Substituting Equations (43) and (44) into Equation (42), the yield condition at this time can be obtained as

(45) $${\sigma_{r}}^{2}+{\sigma_{\theta }}^{2}+\beta \sigma_{r}\sigma_{\theta }+\gamma \left(\sigma_{r}+\sigma_{\theta }\right)={K_{1}}^{2}$$

where $\beta =\frac{\frac{f}{2}-1}{1+\frac{f}{4}}$, $\gamma =\frac{\frac{\alpha K(1-f)}{1+\alpha }}{1+\frac{f}{4}}$, ${K_{1}}^{2}=\frac{\frac{K^{2}}{1+\alpha }{\left(1-f\right)}^{2}}{1+\frac{f}{4}}$.

According to Hooke’s law, the incremental form of the constitutive equation is

(46) $${\dot{\varepsilon }}_{ij}={\dot{\varepsilon }}_{ij}^{e}+{\dot{\varepsilon }}_{ij}^{p}$$

(47) $${\dot{\varepsilon }}_{ij}^{e}=\frac{1}{E}[\left(1+\nu \right){\dot{\sigma }}_{ij}-\nu {\dot{\sigma }}_{kk}\delta_{ij}]$$

where ${\dot{\varepsilon }}_{ij}^{e}$ is the elastic strain rate, ${\dot{\varepsilon }}_{ij}^{p}$ is the plastic strain rate, E is the elastic modulus, and ν is Poisson’s ratio. According to Equation (47), the elastic constitutive relationship is

(48) $${\dot{\varepsilon }}_{r}^{e}=\frac{1}{E}({\dot{\sigma }}_{r}-\nu {\dot{\sigma }}_{\theta })$$

(49) $${\dot{\varepsilon }}_{\theta }^{e}=\frac{1}{E}({\dot{\sigma }}_{\theta }-\nu {\dot{\sigma }}_{r})$$

According to the orthogonal flow rule, the plastic constitutive relation is

(50) $${\dot{\varepsilon }}_{r}^{p}=\lambda [\left(2+\frac{f}{2}\right)\sigma_{r}+\left(\frac{f}{2}-2\right)\sigma_{\theta }+\frac{\alpha K\left(1-f\right)}{1+\alpha }]$$

(51) $${\dot{\varepsilon }}_{\theta }^{p}=\lambda [\left(2+\frac{f}{2}\right)\sigma_{\theta }+\left(\frac{f}{2}-2\right)\sigma_{r}+\frac{\alpha K\left(1-f\right)}{1+\alpha }]$$

Then, according to Equation (46), the incremental form of the final constitutive equation can be obtained as

(52) $${\dot{\varepsilon }}_{r}=\frac{1}{E}\left({\dot{\sigma }}_{r}-\nu {\dot{\sigma }}_{\theta }\right)+\lambda [\left(2+\frac{f}{2}\right)\sigma_{r}+\left(\frac{f}{2}-2\right)\sigma_{\theta }+\frac{\alpha K\left(1-f\right)}{1+\alpha }]$$

(53) $${\dot{\varepsilon }}_{\theta }=\frac{1}{E}\left({\dot{\sigma }}_{\theta }-\nu {\dot{\sigma }}_{r}\right)+\lambda [\left(2+\frac{f}{2}\right)\sigma_{\theta }+\left(\frac{f}{2}-2\right)\sigma_{r}+\frac{\alpha K\left(1-f\right)}{1+\alpha }]$$

### 3.2. Elastic Solutions and Elastoplastic Boundary Conditions

**Figure 3:**
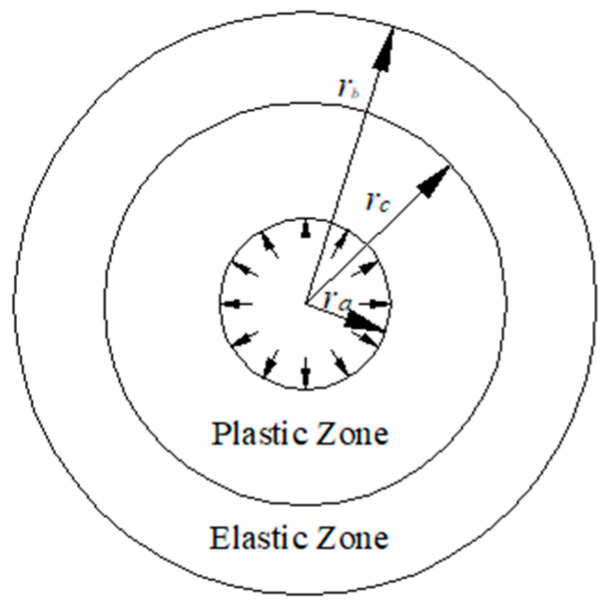
Infinite plate model with a hole in the center.

When an infinite plate with a hole in the center is subjected to the pressure of a parallel plate surface at the orifice, it is an axisymmetric problem of plane stress. Its model is shown in Figure 3, where $r_{a}$ is the inner diameter, $r_{b}$ is the outer diameter, and $r_{c}$ is the elastic–plastic boundary radius.

For this kind of plane stress axisymmetric problem, the equilibrium equation and geometric equation are

(54) $$\frac{d\sigma_{r}}{dr}+\frac{\sigma_{r}-\sigma_{\theta }}{r}=0$$

(55) $$\varepsilon_{r}=\frac{du}{dr},\;\varepsilon_{\theta }=\frac{u}{r}$$

where u=u(r) is the radial displacement, and the compatibility equation is

(56) $$\frac{d}{dr}\left(r\varepsilon_{\theta }\right)=\varepsilon_{r}$$

The stress–strain relationship of the elastic deformation of the material is

(57) $$\varepsilon_{r}=\frac{1}{E}(\sigma_{r}-{\nu \sigma }_{\theta })$$

(58) $$\varepsilon_{\theta }=\frac{1}{E}(\sigma_{\theta }-{\nu \sigma }_{r})$$

From Equations (54), (56), (57), and (58)

(59) $$\left\{\begin{array}{l} \sigma_{r}=\frac{C}{r^{2}}+D\\ \sigma_{\theta }=-\frac{C}{r^{2}}+D \end{array}\right.$$

where *C* and *D* are undetermined constants, at the boundary $r=r_{b}$, $\sigma_{r}=0$. Let the elastic limit load is p, at the elastic–plastic interface $r=r_{c}$, $\sigma_{r}=-p$. Therefore, according to Equation (59), it can be obtained

(60) $$\left\{\begin{array}{c} C=\frac{p{r_{c}}^{2}}{\beta_{1}-1}\\ D=\frac{p\beta_{1}}{1-\beta_{1}} \end{array}\right.$$

where $\beta_{1}={\left(r_{c}/r_{b}\right)}^{2}$, and at the elastic–plastic interface $r=r_{c}$, there is

(61) $$\left\{\begin{array}{l} \sigma_{r}=-p\\ \sigma_{\theta }=p(\frac{1+\beta_{1}}{1-\beta_{1}}) \end{array}\right.$$

For plane stress problems, when $r=r_{c}$, the stress should satisfy the yield condition Equation (45). By substituting Equation (61) into (45), the elastic limit load for plane stress problems can be obtained

(62) $$p=\frac{\left(\sqrt{\gamma^{2}{\beta_{1}}^{2}+\beta_{2}{K_{1}}^{2}}-\gamma \beta_{1}\right)\left(1-\beta_{1}\right)}{\beta_{2}}$$

where $\beta_{2}=\left(2+\beta \right){\beta_{1}}^{2}+2-\beta$.

Under the monotonic increasing internal pressure, when the load exceeds the elastic limit of the material, the inner diameter of the hole enters the plastic deformation stage from elastic deformation, and with the increase in the load, the elastic–plastic boundary radius $r_{c}$ also increases. To determine the displacement field of the material deformation process, the Prager hypothesis [30,31] is introduced in the plastic deformation zone, so that

(63) $$m=\frac{r_{c}}{r},r_{a}\leq r\leq r_{c}$$

The parameter *m* is used to record the plastic deformation history. In the plastic zone, under the Prayer hypothesis, the form of “rate” can also be expressed by *m*, i.e.,

(64) $$\frac{d}{dt}\left(\;\right)=\frac{d}{dm}\left(\;\right)$$

(65) $$\frac{d}{dr}\left(\;\right)=\frac{dm}{dr}\frac{d}{dm}\left(\;\right)=-\frac{m^{2}}{r_{c}}\frac{d}{dm}\left(\;\right)$$

According to Equations (55), (57), and (58), the

(66) $$u=\frac{r}{E}\frac{p{r_{c}}^{2}}{1-\beta_{1}}\left[\frac{1}{r^{2}}\left(1+\nu \right)+\frac{1}{{r_{b}}^{2}}\left(1-\nu \right)\right]$$

(67) $$\varepsilon_{r}=-\frac{p}{E\left(1-\beta_{1}\right)}\left[m^{2}\left(1+\nu \right)+\beta_{1}\left(\nu -1\right)\right]$$

(68) $$U=\frac{u}{r_{c}}=\frac{p}{E\left(1-\beta_{1}\right)}\left[m\left(1+\nu \right)+\frac{\beta_{1}\left(1-\nu \right)}{m}\right]$$

According to Equation (65), the

(69) $$\frac{du}{dr}=-\frac{m^{2}}{r_{c}}\frac{du}{dm}=-m^{2}\frac{dU}{dm}$$

(70) $$\frac{dU}{dm}=\frac{p}{E\left(1-\beta_{1}\right)}\left[\left(1+\nu \right)+\frac{\beta_{1}\left(\nu -1\right)}{m^{2}}\right]$$

When *m* = 1, the displacement boundary condition is

(71) $$U=\frac{p}{E\left(1-\beta_{1}\right)}\left[\left(1+\nu \right)+\beta_{1}\left(1-\nu \right)\right]$$

(72) $$\frac{dU}{dm}=\frac{p}{E\left(1-\beta_{1}\right)}\left[\left(1+\nu \right)+\beta_{1}\left(\nu -1\right)\right]$$

For an infinite plate with a hole in the center, when $r_{b}\to \infty$, $\beta_{1}=0$. At this time, the elastic–plastic limit load is

(73) $$p=\frac{\sqrt{\beta_{2}}}{\beta_{2}}K_{1}$$

Therefore, the boundary conditions of the stress field and displacement field at the elastic–plastic interface are

(74) $$\sigma_{r}=-p$$

(75) $$\sigma_{\theta }=p$$

(76) $$U=\frac{p\left(1+\nu \right)}{E}$$

(77) $$\frac{dU}{dm}=\frac{p\left(1+\nu \right)}{E}$$

### 3.3. Elastoplastic Solution

According to Equations (52), (53), (54), (64), and (65), the equilibrium equation expressed by parameter *m* is

(78) $$m\frac{d\sigma_{r}}{dm}+\sigma_{\theta }-\sigma_{r}=0$$

In the elastic–plastic region, the constitutive equation is

(79) $$\frac{d\varepsilon_{r}}{dm}=\frac{1}{E}\left(\frac{d\sigma_{r}}{dm}-\nu \frac{d\sigma_{\theta }}{dm}\right)+\lambda \left[\left(2+\frac{f}{2}\right)\sigma_{r}+\left(\frac{f}{2}-2\right)\sigma_{\theta }+\frac{\alpha K\left(1-f\right)}{1+\alpha }\right]$$

(80) $$\frac{d\varepsilon_{\theta }}{dm}=\frac{1}{E}\left(\frac{d\sigma_{\theta }}{dm}-\nu \frac{d\sigma_{r}}{dm}\right)+\lambda \left[\left(\frac{f}{2}-2\right)\sigma_{r}+\left(2+\frac{f}{2}\right)\sigma_{\theta }+\frac{\alpha K\left(1-f\right)}{1+\alpha }\right]$$

According to the geometric equation and Equation (46), the incremental form of the constitutive equation can be written as the expression of dimensionless displacement *U*

(81) $${\dot{\varepsilon }}_{r}={\dot{\varepsilon }}_{r}^{e}+{\dot{\varepsilon }}_{r}^{p}=\frac{d}{dm}\left(\frac{du}{dr}\right)=-m^{2}\frac{d^{2}U}{dm^{2}}-2m\frac{dU}{dm}$$

(82) $${\dot{\varepsilon }}_{\theta }={\dot{\varepsilon }}_{\theta }^{e}+{\dot{\varepsilon }}_{\theta }^{p}=\frac{d}{dm}\left(\frac{u}{r}\right)=m\frac{dU}{dm}+U$$

Order

(83) $$\frac{1}{E}\left(\frac{d\sigma_{r}}{dm}-\nu \frac{d\sigma_{\theta }}{dm}\right)=F_{1}\left(\frac{d\sigma_{r}}{dm},\frac{d\sigma_{\theta }}{dm}\right)$$

(84) $$\left(2+\frac{f}{2}\right)\sigma_{r}+\left(\frac{f}{2}-2\right)\sigma_{\theta }+\frac{\alpha K\left(1-f\right)}{1+\alpha }=F_{2}\left(\sigma_{r},\sigma_{\theta },\alpha \right)$$

(85) $$\frac{1}{E}\left(\frac{d\sigma_{\theta }}{dm}-\nu \frac{d\sigma_{r}}{dm}\right)=F_{3}\left(\frac{d\sigma_{r}}{dm},\frac{d\sigma_{\theta }}{dm}\right)$$

(86) $$\left(\frac{f}{2}-2\right)\sigma_{r}+\left(2+\frac{f}{2}\right)\sigma_{\theta }+\frac{\alpha K\left(1-f\right)}{1+\alpha }=F_{4}\left(\sigma_{r},\sigma_{\theta },\alpha \right)$$

According to Equations (79)–(86), *λ* can be eliminated to obtain

(87) $$F_{4}\left(m^{2}\frac{d^{2}U}{dm^{2}}+2m\frac{dU}{dm}\right)+F_{2}\left(m\frac{dU}{dm}+U\right)=F_{2}F_{3}-F_{1}F_{4}$$

Thus, the quadratic nonlinear differential equation of dimensionless displacement *U* can be obtained

(88) $$F_{4}m^{2}\frac{d^{2}U}{dm^{2}}+\left(2mF_{4}+mF_{2}\right)\frac{dU}{dm}+F_{2}U=F_{2}F_{3}-F_{1}F_{4}$$

Both sides of the yield condition Equation (45) are differentiated at the same time

(89) $$2\sigma_{r}\frac{d\sigma_{r}}{dm}+2\sigma_{\theta }\frac{d\sigma_{\theta }}{dm}+\beta \sigma_{\theta }\frac{d\sigma_{r}}{dm}+\beta \sigma_{r}\frac{d\sigma_{\theta }}{dm}+\gamma \left(\frac{d\sigma_{r}}{dm}+\frac{d\sigma_{\theta }}{dm}\right)=0$$

According to Equation (78), it can be obtained that

(90) $$\frac{d\sigma_{r}}{dm}=\frac{\sigma_{r}-\sigma_{\theta }}{m}$$

Therefore, it can be concluded that

(91) $$\frac{d\sigma_{\theta }}{dm}=\frac{2\sigma_{r}+\beta \sigma_{\theta }+\gamma }{2\sigma_{\theta }+\beta \sigma_{r}+\gamma }\left(\frac{\sigma_{\theta }-\sigma_{r}}{m}\right)$$

To ensure the convexity of the loading surface, the plastic flow factor λ in the plastic zone should meet

(92) λ≥0

Next, calculate the plastic flow factor *λ*. Note that

(93) $$m\frac{d}{dm}\left(m\frac{dU}{dm}+U\right)=m^{2}\frac{d^{2}U}{dm^{2}}+2m\frac{dU}{dm}$$

Therefore, according to Equations (80) and (82), it can be obtained that

(94) $$m^{2}\frac{d^{2}U}{dm^{2}}+2m\frac{dU}{dm}=\frac{m}{E}\left(\frac{d^{2}\sigma_{\theta }}{dm^{2}}-\nu \frac{d^{2}\sigma_{r}}{dm^{2}}\right)+m\frac{d\lambda }{dm}\left[\left(\frac{f}{2}-2\right)\sigma_{r}+\left(2+\frac{f}{2}\right)\sigma_{\theta }+\frac{\alpha K\left(1-f\right)}{1+\alpha }\right]+m\lambda \left[\left(\frac{f}{2}-2\right)\frac{d\sigma_{r}}{dm}+\left(2+\frac{f}{2}\right)\frac{d\sigma_{\theta }}{dm}\right]$$

Combining Equations (79) and (81), we can get that the differential equation for λ is

(95) $$m\frac{d\lambda }{dm}\left[\left(\frac{f}{2}-2\right)\sigma_{r}+\left(2+\frac{f}{2}\right)\sigma_{\theta }+\frac{\alpha K\left(1-f\right)}{1+\alpha }\right]+\lambda \left[m\left(\frac{f}{2}-2\right)\frac{d\sigma_{r}}{dm}+m\left(2+\frac{f}{2}\right)\frac{d\sigma_{\theta }}{dm}+\left(2+\frac{f}{2}\right)\sigma_{r}+\left(\frac{f}{2}-2\right)\sigma_{\theta }+\frac{\alpha K\left(1-f\right)}{1+\alpha }\right]\;+\frac{1}{E}\left(\frac{d\sigma_{r}}{dm}-\nu \frac{d\sigma_{\theta }}{dm}+m\frac{d^{2}\sigma_{\theta }}{dm^{2}}-m\nu \frac{d^{2}\sigma_{r}}{dm^{2}}\right)=0$$

No plastic strain under elastic and unloading conditions, *λ* = 0, then at the elastic–plastic interface, there is

(96) $${\left.\lambda \right|}_{m=1}=0$$

## 4. Numerical Calculation Results and Discussion

**Figure 4:**
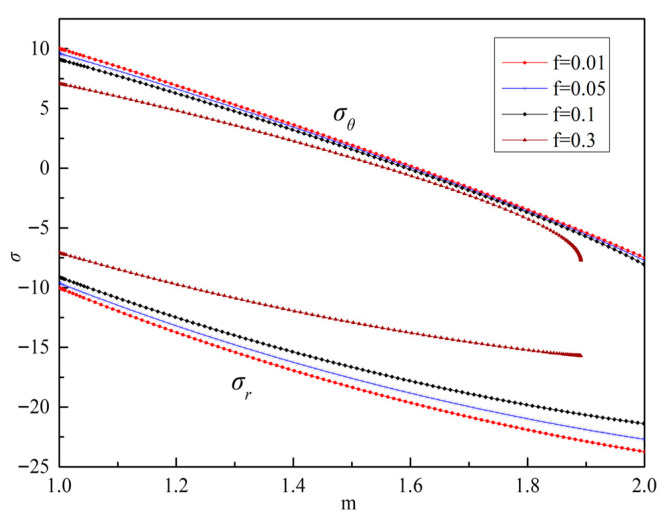
Numerical solution of stress with different *f*.

**Figure 5:**
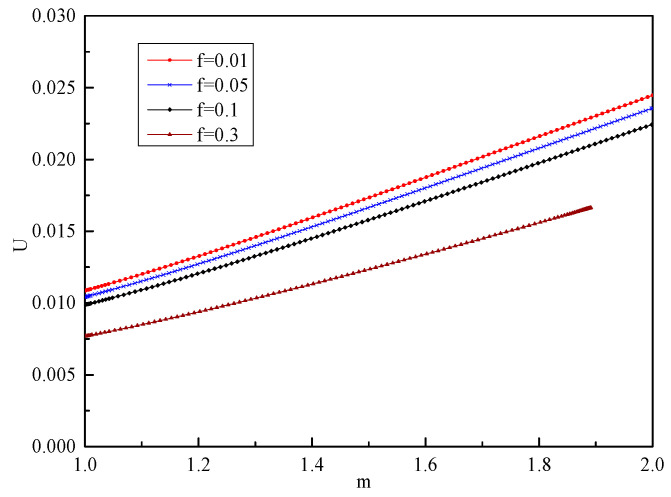
Numerical solution of *U* with different *f*.

**Figure 6:**
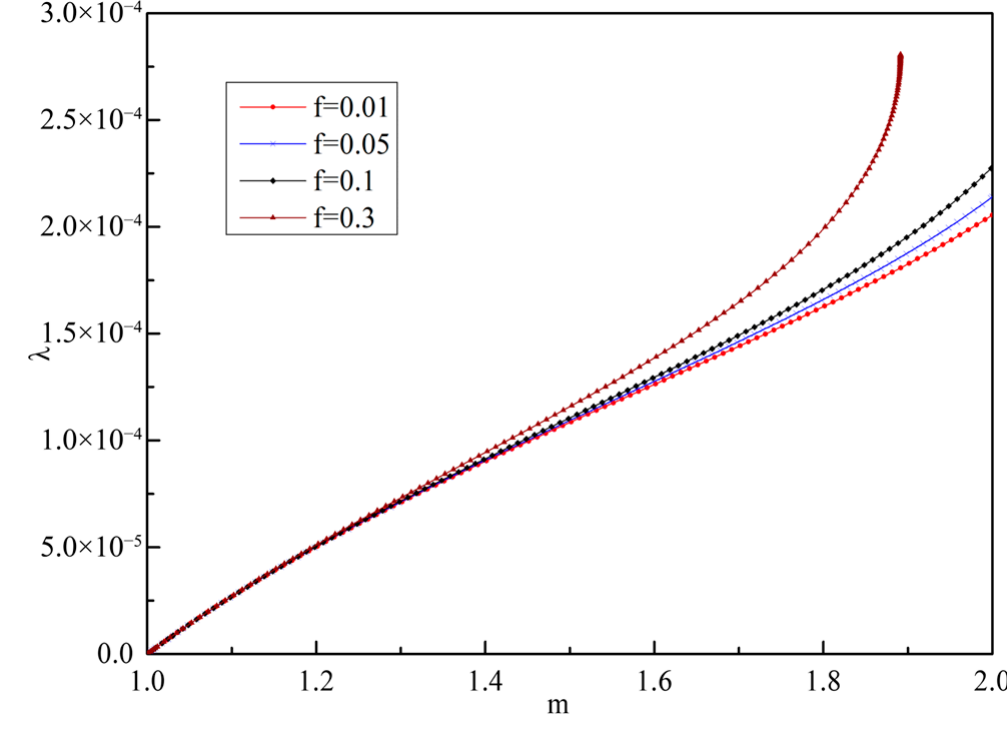
Numerical solution of *λ* with different *f*.

According to the boundary conditions given in Equation (66), considering the influence of different void volume fractions on stress and displacement, the differential equation is solved by using the material parameters in Table 1, and the numerical solutions of stress, displacement, and plastic flow factor under different void volume fraction are obtained, as shown in Figure 4, Figure 5 and Figure 6.

It can be seen from Figure 4 that with the increase in *m*, that is, the closer to the orifice, the absolute value of $\sigma_{r}$ increases, while $\sigma_{\theta }$ changes relatively, and the orifice first enters the yield state and is damaged. In addition, $\sigma_{r}$ is obviously affected by *f*. With the increase in *f*, $\sigma_{r}$ decreases, and the elastic limit load *p* of the material also decreases. It can be seen from Figure 5 that the closer to the orifice, the greater the displacement, and the hole continues to expand outward. With the increase in *f*, the displacement at the orifice decreases, which is similar to the conclusion in reference [30]. However, considering the different basic yield models of the two, the results of the numerical solution are significantly different. The numerical results presented in this paper show that the variation patterns of stress and displacement fields are similar to those reported in Ref. [6]. However, the material parameters used in Ref. [6] are tensile and compressive yield strengths, and the discussion focuses on the variation patterns of stress and displacement fields corresponding to the ratio of these two material parameters, without considering the impact of damage on deformation. In contrast, this paper defines two material parameters using yield strength and Poisson’s ratio. Since the stress field is not normalized, the discussion is limited to the influence of different void volume fractions on field variables when Poisson’s ratio is 0.3. It can be seen from Figure 6 that the closer to the orifice, the faster the material flows.

It is also found that when *f* = 0.3, the numerical solution results are quite different, and when *m* = 1.9, the numerical solution will no longer converge. The reason for this may be that under the current void volume fraction, due to the relatively high void volume fraction, after loading, the voids coalesce earlier, and the structural deformation tends to localize earlier, resulting in the cessation of elastoplastic boundary extension. At the same time, it may also be affected by parameter definitions. When defining the material parameters *K* and *α* in the model, the paper directly uses the yield strength and Poisson’s ratio of the material. Although the yield surface described in Equation (41) has similar laws to the GTN model and the Lee model, *α* is directly defined as Poisson’s ratio, which leads to the fact that the meso-damage model proposed in the paper cannot be simplified to the GTN model under certain circumstances. The material parameter *α* should be more inclined to the functional relationship of Poisson’s ratio. When the Poisson’s ratio of the material is 0.5, Equation (41) can be transformed into the GTN model, and the solution result should be more accurate. Future research work can be carried out in this direction. At the same time, if the stress can also be dimensionless, then the influence of different Poisson’s ratios on the field quantity can also be discussed when the hole volume fraction is fixed.

## 5. Conclusions

The deformation of plastic porous media is inevitably affected by the volume fraction of holes. The plane stress problem of such materials is analyzed by using the meso-damage model, and the following conclusions are obtained:

(1) Based on the parabolic two-parameter yield function, a meso-damage model including damage is established by introducing the void volume fraction through theoretical derivation. The parameters *K* and *α* in the model are defined by the material yield strength and Poisson’s ratio, respectively. The yield surface of the model is consistent with that of the existing meso-damage model.
(2) The constitutive equation of the plane stress problem is established by using the meso-damage model, and the self-similarity assumption is introduced. Combined with the equilibrium equation and geometric equation of the plane stress problem, the differential equations for solving the stress field, displacement field, and plastic flow factor are obtained. The numerical results show that when *f* is constant, the closer to the orifice, the greater the radial stress, the greater the displacement, the faster the material flow, and the orifice preferentially enters the yield state. With the increase in *f*, the radial stress and displacement decrease, while the change in circumferential stress is relatively small. The flow of the material under different *f* has an obvious difference near the orifice, but has little change far away from the orifice.
(3) The definition of parameter *α* in the paper only considers the influence between volume deformation and Poisson’s ratio, and directly defines the value of *α* as equal to Poisson’s ratio, which severely limits the application of this model. If the expression between α and Poisson’s ratio can be obtained through rigorous theoretical analysis, it will enhance the effectiveness of the model and further expand its application scope.
(4) In the process of plastic deformation, the evolution of pores is influenced by the stress state (for example, under high stress triaxiality, the volume of pores will increase, and under a shear stress state, the shape of pores will undergo significant changes), but the paper does not mention how the evolution of pores occurs during plastic deformation. Future research could consider introducing an evolution function of pore volume fraction, coupled with experimental verification, which would quantify the differences between the calculated results of the model and the experiments and could improve the credibility or direction of improvement of the model.

## Figures and Tables

**Table 1:** Parameters of aluminum foam [32].

*E*/MPa	ν	$\sigma_{s}$/MPa
1.2 × 10^3^	0.3	20

## Data Availability

The original contributions presented in this study are included in the article; further inquiries can be directed to the corresponding author.

## Nomenclature

*K*, *α*	Material parameter
${\dot{\varepsilon }}_{ij}$	Strain rate
$s_{ij}$	Microscopic deviatoric stress tensor
$\sigma_{kk}$	The first invariant of the stress tensor
$\sigma_{ij}$	Stress tensor
$\delta_{ij}$	Kroneck symbol
${\dot{e}}_{ij}$	Strain rate deviatoric tensor
λ	Plastic flow factor
${\dot{\varepsilon }}_{kk}$	Strain rate spherical tensor
$\bar{\sigma }$	Mises equivalent stress
$\dot{\bar{\varepsilon }}$	Mises equivalent strain rate
$\dot{w}$	Plastic dissipation power
${\dot{\varepsilon }}_{ij}^{V}$	The volume change part of the meso-strain rate
${\dot{\varepsilon }}_{ij}^{S}$	The shape change part of the meso-strain rate
$v_{i}$	Microscopic velocity field
$v_{i}^{V}$	The volume change part of the microscopic velocity field
$v_{i}^{S}$	The shape change part of the microscopic velocity field
${\dot{\varepsilon }}_{rr}$,${\dot{\varepsilon }}_{\theta \theta }$,${\dot{\varepsilon }}_{\phi \phi }$	Strain rates in three directions in spherical coordinate system
${\dot{E}}_{ij}$	Macroscopic strain rate
$X_{j}$	The macroscopic velocity field in Cartesian coordinate
S	The unit cube outer surface
$a_{0}$	Meso-volume strain rate
*a*, *b*	Inner and outer radius of void-matrix model
${\dot{E}}_{ij}^{\prime }$	Macroscopic strain rate deviatoric tensor
*r*	Radius of a point inside the matrix
$x_{i}$	Microscopic position vector, Cartesian coordinates
$n_{i}$	Unit normal vector on S
$h_{ij}$	Structure tensor
V	Volume of the unit cell
$\Sigma_{ij}$	Macroscopic stress tensor
$\dot{W}$	Macroscopic dissipation
$\Sigma_{ij}^{\prime }$	Macroscopic deviatoric stress tensor
$\Sigma_{nn}$	Macroscopic dilatational stress, spherical model
$\Sigma_{eq}$	Macroscopic equivalent stress
*f*	Void volume fraction
$\sigma_{r}$, $\sigma_{\theta }$	Radial and circumferential stress
*E*	Young’s modulus
${\dot{\varepsilon }}_{ij}^{e}$, ${\dot{\varepsilon }}_{ij}^{p}$	Elastic and plastic strain rate
$\varepsilon_{r}$, $\varepsilon_{\theta }$	Radial and circumferential strain
ν	Poisson’s ratio
${\dot{\varepsilon }}_{r}$, ${\dot{\varepsilon }}_{\theta }$	Radial and circumferential strain rate
*u*	Radial displacement
$r_{a}$, $r_{b}$, $r_{c}$	Inner radius, outer radius, elastic–plastic boundary radius
*p*	Elastic limit load
*U*	Dimensionless displacement

## References

[1] Kirsch E.G. (1898). Die theorie der elastizität und die bedürfnisse der festigkeitslehre. Z. Des Vereines Dtsch. Ingenieure.

[2] Inglis C.E. (1913). Stresses in a plate due to the presence of cracks and sharp corners. Proc. Inst. Naval. Arch..

[3] Westergaard H.M. (1939). Bearing pressures and cracks. ASME J. Appl. Mech..

[4] Irwin G.R. (1957). Analysis of Stresses and Strains Near the End of a Crack Traversing a Plate. J. Appl. Mech..

[5] Guo R.P., Liu G.T., Fan T.Y. (2009). Review of Compressible Plasticity Mechanics in Metal Foams. Rare Met. Mater. Eng..

[6] Tang L.Q., Wang Y.H. (1990). Elastic-plastic solution for an axisymmetric plane stress problem in MVM material. J. Harbin Shipbuild. Eng. Inst..

[7] Bijak-Zochowski M., Marek P. (1996). Development of plastic zones and residual stress in elasto-plastic contact problems with stress singularities in elastic range. Int. J. Mech. Sci..

[8] Zhou W.X., Zhou X. (1996). An analytic solution for axisymmetric plane stress problem of plastic anisotropic materials considering strain hardening. Shanghai J. Mech..

[9] Li T., Zhang Q.H. (2002). The characteristic field of plane stress problem for the Orthotropic materials under R. Hill yield criterion (h > 2). Eng. Mech..

[10] Liu J., Wang D.J. (2006). Research on determing criterion of deformation stability for metal plasticity. J. Plast. Eng..

[11] Li Y.Y., Dan Y., Cai R.X. (2009). Axisymmetrical 2-D Analytical Solutions of Ideal Plasticity. J. Mech. Eng..

[12] Liu M.W., Gao Y.H., Zhang D.P., Wang Z.X., Lei Y.J. (2021). Elastic-plastic analysis for thermoplastic composite plane curved beams by bending moment. J. Mech. Strength.

[13] Roman J. (2024). Approximate solution of plane problems about stress concentrations in elastic bodies with voids. J. Eng. Math..

[14] Chen H.L., Jiang W., Benjamin W.S. (2024). A mixed formulation of the plane-stress problem to facilitate reuse of constitutive models in finite-element programs. Mech. Res. Commun..

[15] Lyamina E.A. (2024). A General Axisymmetric Elastic-Plastic Solution for an Arbitrary Isotropic Yield Criterion under Plane Stress. Mech. Solids.

[16] Li W.M., Qiu P., Luo J.T. (2011). Displacement Solution of Axisymmetrical Plane Stress Problem in Plastic State. J. Mech. Eng..

[17] Qin S.J., Yang L., Gai B.B. (2014). Analyses of Wrinkling in Flange Region and Rupture in Axisymmetric Deep Drawing under Plane Stress Model. China Mech. Eng..

[18] Giginyak F.F., Bulakh P.A. (2015). Influence of Preliminary Deformation on the Characteristics of Cyclic Creep and Damageability of Steel 10GN2MFA Under Plane Stress Conditions. Strength Mater..

[19] Qin S.J., Kong X.H., Yang L. (2019). Direct integration method for solving axisymmetric sheet forming problem based on incremental theory. China Mech. Eng..

[20] Woelke P.B. (2020). Simplification of the Gurson model for large-scale plane stress problems. Int. J. Plast..

[21] Dan G. (2021). Asymptotic Analysis for Plane Stress Problems. J. Elast..

[22] Mu Z.K., Zhao J., Meng Q.D., Zhang Y., Yu G.C. (2022). Limitation analysis of the Hill48 yield model and establishment of its modified model for planar plastic anisotropy. J. Mater. Process. Technol..

[23] Bulakh P.A., Maslo O.M. (2023). A study of the deformation properties of 10GN2MFA and 15Kh2MFA steels taking into account material damageability under plane stress conditions. Strength Mater..

[24] Zhu H.H., Lin Y.L., Chen K.L., He Z.B., Yuan S.J. (2023). A Homogeneous Anisotropic Hardening Model in Plane Stress State for Sheet Metal under Nonlinear Loading Paths. Materials.

[25] Christensen R.M. (2004). A Two-Property Yield, Failure (Fracture) Criterion for Homogeneous, Isotropic Materials. J. Eng. Mater. Technol..

[26] Christensen R.M. (2006). A Comparative Evaluation of Three Isotropic, Two Property Failure Theories. J. Appl. Mech..

[27] Rice J.R., Tracey D.M. (1969). On the ductile enlargement of voids in triaxial stress fields. J. Mech. Phys. Solids.

[28] Lee J.H., Oung J. (2000). Yield functions and flow rules for porous pressure-dependent strain-hardening polymeric materials. J. Appl. Mech..

[29] Gurson A.L. (1977). Continuum theory of ductile rupture by void nucleation and growth: Part I-yield criteria and flow rules for porous ductile media. J. Eng. Mater. Technol..

[30] Zhang X.Y. (2009). Meso-Analysis and Application of Yield Function of Pressure-Sensitive Materials. Master’s Thesis.

[31] Prager W., Hodge P.G. (1964). Theory of Perfectly Plastic Solids.

[32] Xin C.L., Xue Z.Q., Tu J., Wang X.Q., Sun F.T., Shi D.Y. (2019). Handbook of Common Material Parameters for Finite Element Analysis.

